# The Terrestrial Plastisphere: Diversity and Polymer-Colonizing Potential of Plastic-Associated Microbial Communities in Soil

**DOI:** 10.3390/microorganisms9091876

**Published:** 2021-09-03

**Authors:** Joana MacLean, Sathish Mayanna, Liane G. Benning, Fabian Horn, Alexander Bartholomäus, Yosri Wiesner, Dirk Wagner, Susanne Liebner

**Affiliations:** 1 GFZ German Research Centre for Geosciences, Section Geomicrobiology, 14473 Potsdam, Germany; gfz.fabian@gmail.com (F.H.); abartho@gfz-potsdam.de (A.B.); dirk.wagner@gfz-potsdam.de (D.W.); sliebner@gfz-potsdam.de (S.L.); 2 GFZ German Research Centre for Geosciences, Section Interface Geochemistry, 14473 Potsdam, Germany; sathish.mayanna@gmail.com (S.M.); liane.g.benning@gfz-potsdam.de (L.G.B.); 3 Carl Zeiss Microscopy GmbH, Carl Zeiss Strasse 22, 73447 Oberkochen, Germany; 4 Department of Earth Sciences, Free University of Berlin, 12249 Berlin, Germany; 5 Department of Physical and Chemical Analysis of Polymers, BAM Berlin, 12205 Berlin, Germany; yosri.wiesner@bam.de; 6 Institute of Geosciences, University of Potsdam, 14476 Potsdam, Germany; 7 Institute of Biochemistry and Biology, University of Potsdam, 14476 Potsdam, Germany

**Keywords:** plastisphere, plastic pollution, soil microbial community, microbial diversity, biofilms, microbe–plastic interactions, polyethylene colonization, FESEM imaging

## Abstract

The concept of a ‘plastisphere microbial community’ arose from research on aquatic plastic debris, while the effect of plastics on microbial communities in soils remains poorly understood. Therefore, we examined the inhabiting microbial communities of two plastic debris ecosystems with regard to their diversity and composition relative to plastic-free soils from the same area using 16S rRNA amplicon sequencing. Furthermore, we studied the plastic-colonizing potential of bacteria originating from both study sites as a measure of surface adhesion to UV-weathered polyethylene (PE) using high-magnification field emission scanning electron microscopy (FESEM). The high plastic content of the soils was associated with a reduced alpha diversity and a significantly different structure of the microbial communities. The presence of plastic debris in soils did not specifically enrich bacteria known to degrade plastic, as suggested by earlier studies, but rather shifted the microbial community towards highly abundant autotrophic bacteria potentially tolerant to hydrophobic environments and known to be important for biocrust formation. The bacterial inoculates from both sites formed dense biofilms on the surface and in micrometer-scale surface cracks of the UV-weathered PE chips after 100 days of in vitro incubation with visible threadlike EPS structures and cross-connections enabling surface adhesion. High-resolution FESEM imaging further indicates that the microbial colonization catalyzed some of the surface degradation of PE. In essence, this study suggests the concept of a ‘terrestrial plastisphere’ as a diverse consortium of microorganisms including autotrophs and other pioneering species paving the way for those members of the consortium that may eventually break down the plastic compounds.

## 1. Introduction

Plastics are known for being remarkably resistant to natural degradation processes. An attribute most favorable in the first place, has ultimately resulted in one of the most disastrous environmental calamities of our times. Through their versatile size and materiality, plastic debris entered habitats of many organisms, and recent research has identified a number of adverse effects on (macro-) fauna and flora in pristine environments [1,2].

Fibers and fragments of synthetic polymers (plastics) were shown to influence the assemblage of microbial taxa present in their proximity. This ecological phenomenon was recently referred to as the concept of a ‘plastisphere microbial community’ [3]. The ‘plastisphere’ was first suggested to be a distinct microbial habitat by studies carried out on aquatic plastic debris in the North Atlantic Ocean. This work identified relevant microbial inhabitants of plastic debris, and furthermore opened up a broader discourse about plastic waste as a habitat-forming agent [4,5,6]. All plastics found in the ocean were initially produced and handled on land. Plastic enters and accumulates terrestrial ecosystems due to improper disposal, blow-offs from landfills, remains of mulching foils and other plastics used in agricultural applications in fields [7]. Newer studies estimated the amount of microplastic (particle size between 1 μm and 5 mm) in soil to be 4–23 times higher than in the ocean. This number stresses the relevance to study the impacts of plastics as an ecological agent in soil [7,8]. Nevertheless, the occurrence and ecological impact of plastic debris has still been studied almost exclusively in aquatic ecosystems, whereas research on dynamics and extent of plastic pollution in terrestrial habitats is still in short supply [9,10,11].

In soil, microorganisms are involved in the degradation processes of both natural and synthesized material, generally making them the first responders to environmental perturbation [12]. Along with soil geochemical factors that shape the microbial community, studies showed a sensitive response of the soil microbiota in areas subjected to oil spillages and other anthropogenic contamination [13]. The community composition of sediment-dwelling bacteria and their diversity was found to be shaped by contaminants (metals and polycyclic aromatic hydrocarbons), and the abundant core set of bacteria was held responsible for the community response to contamination [14]. Following the assumption that microbial communities in terrestrial habitats are substrate-dependent and influenced by their surrounding habitat [3], we hypothesize that plastic debris forms a selective habitat in soil in which the community structure is distinct from that of soil without a substantial plastic content. To our knowledge, there are in total six studies which examined plastic-associated microbial communities in soil using a next-generation sequencing approach [15,16,17,18,19,20]. While most of these studies found the plastic-associated microbial community to be less diverse when compared to the surrounding bulk soil [17,18,19,20], two studies reported a distinct microbial community on plastic debris in which a plastic-specific microbiota is enriched from the soil microbial community by the presence of plastic [15,16]. Thus, how far soil-buried plastic debris selects for a specific microbial community composition still remains poorly understood.

Besides ecological studies addressing the concept of a ‘microbial plastisphere’, the aim to discover microorganisms capable of degrading synthetic polymers has motivated a number of microbial studies in the last few years [15,16,17,18,19,20,21,22,23,24,25,26,27]. So far, microorganisms from more than 90 genera were found to possess plastic-degrading abilities [22]. Those findings created a scientific framework for future eco-remediation technologies. The biodegradation of plastics is a complex and successional process which consists of several steps and often involves the presence of a diverse and multispecies consortium of microorganisms [27]. Once discarded on the ground, plastic becomes fragmented into smaller pieces by mechanical forces, and its surface properties are altered over time, in particular through UV radiation, fluctuating moisture regimes, and high temperature. In order to finally degrade these complex chemical compounds, microorganisms must act in close proximity with the hydrophobic polymer surface. Biofilms formed by microbial cells embedded in extracellular polymeric substances (EPS) were shown to colonize and degrade hydrophobic substrates much better than single planktonic cells [28]. Studies that examined the biodegradation of plastics in soils observed that the surface fragmentation of buried polyethylene (PE) sheets was enhanced proportionally to the growth of biofilms on the PE surface [29]. Therefore, we postulate that plastic debris in soil is not only inhabited by distinct microbial communities relative to natural soils in which plastic does not have a major contribution to the soil matrix. We also expect that soil-buried plastic debris hosts microbial communities that can colonize and attach to the hydrophobic polymer surface as an important prerequisite for the complete biodegradation of common plastic types.

In order to address these hypotheses, we sampled plastic debris of a plastic recycling factory and an abandoned landfill and characterized the microbial communities inhabiting them using 16S rRNA amplicon sequencing. Soil material from the same sampling locations were sequenced in order to distinguish a plastic-associated microbiome from the local soil microbial community. Furthermore, the microbial communities from plastic debris were tested for their plastic-colonizing potential on UV-weathered and unweathered PE chips using field emission scanning electron microscopy (FESEM) imaging. With this, our work adds to the few recent studies combining molecular approaches with advanced imaging techniques to characterize microbial taxa in plastic-polluted ecosystems [30,31].

## 2. Materials and Methods

### 2.1. Sampling

Plastic debris was sampled at two independent geographical locations characterized by plastic particles of different type and origin (Supplementary Figures S3 and S4). By including samples of adjacent soils that had a negligible plastic content, we aimed to identify taxa that occurred preferably on plastic debris, while at the same time excluding a geographical location effect. Our plastic debris samples contained plastic particles, foils and fibers (2.9%–43.5% mass percentage of the soil mass), whereas the reference soil samples consisted of soil that had no apparent plastic particles present and showed a total plastic content of less than 1% of the total soil mass (Supplementary Figure S1).

A first set of samples was collected in January 2017 at seven different sampling spots on an abandoned landfill at Niemegk, a town in the Potsdam-Mittelmark district of Brandenburg, north-eastern Germany (52°02′58.8″ N 12°39′34.8″ E). The plastic debris samples (NGK P1.3–NGK P18) were collected directly from plastic waste on the landfill at five different spots. The reference soil samples (NGK S1.1, NGK S1.2, NGK S18.1 and NGK S18.7) were retrieved from three sites between 10 and about 50 m distance from the landfill from adjacent meadow soil.

The second sampling site was a plastic recycling factory near Eisenhüttenstadt, a town in the Oder-Spree district of the state of Brandenburg, Germany (52°09′44.1″ N 14°37′02.6″ E). The factory grounds and the surrounding area were visited in September 2017 with the kind permission of ALBA Recycling GmbH. Plastic debris (Alba P9.1 and Alba P9.2) was collected from one spot adjacent to the factory’s building, whereas reference soil samples (Alba S7.1, Alba 7.2 and Alba S8.1, Alba 8.2) were collected at two different sampling spots from the upper soil layer of a surrounding meadow located at 76 and 100 m distance from the factory.

All samples were sealed in sterile plastic bags (LDPE), put on ice for transportation and immediately stored in −20 °C until further processing.

### 2.2. Microbial Community Analysis

#### 2.2.1. DNA Extraction

DNA extraction from both sample types was carried out using the Roboklon EurX Soil DNA Purification Kit. Per sample 270–380 mg of material was used for DNA extraction procedure. A slight alteration from the protocol was implemented in the initial five steps of the protocol: in order to homogenize the hardest plastic pieces and to optimize DNA yield, the provided EurX Buffer for cell lysis was transferred to the FastPrep^®^ Bead Tubes so that the FastPrep^®^ Cell disrupter could be used for 45 s twice at a speed setting of 5.5. Subsequent steps were performed following the manufacturer guidelines, and the samples were eluted in 75 µL elution buffer. The DNA concentrations were measured using a Qubit^®^ Fluorometer 2.0. After a first quality check on 1% agarose gel, gDNA samples were treated with Thermo Fisher™ RNAse A to remove RNA contamination prior to sequencing.

#### 2.2.2. Paired End Illumina Sequencing and Bioinformatics

After a first general amplification of 16S rRNA gene sequences using the universal primers for bacteria 341F(5′ CCT ACG GGA GGC AGC AG 3′) and 805R (5′ GAC TAC HVGGT ATC TAA TCC 3′), primer pairs including specific barcodes were assigned to each of the PCR products in order to identify each sample from the pooled library. Prior to sequencing, all PCR products were purified using a magnetic beads Agencourt^®^ AMPure^®^ XP- Kit (Beckman Coulter Life Sciences, Krefeld, Germany) according to the protocol. Illumina paired end sequencing was performed at Eurofins Genomics (Ebersberg, Germany) on an Illumina HiSeq machine with MiSeq V3 chemistry (2 × 300bp paired-end reads). PhiX control v3 library was included with 15% for better performance due to different sequencing length. The sequencing library was demultiplexed using Cutadapt [32]. A 10% sequencing error was allowed in the primer sequence, while no error mismatch was allowed in the barcode sequences. It was ensured that the barcode sequence quality score was above Q25. The DADA2 workflow [33] was applied for further sequence analysis. The sequences were truncated (250 bp forward read; 200 bp reverse read) and quality filtered. An error-model was generated for each library and sequences were dereplicated, error-corrected and merged. Sequences with a minimum length of 200 bp were used for the construction of a sequence table, which was further checked for chimeras using the de novo method [33]. The resulting amplicon sequence variants (ASVs) were taxonomically assigned using the SILVA taxonomy database (v132) [34] with the help of vsearch [35], as provided in the QIIME2 pipeline [36]. Raw sequencing data are available at the European Nucleotide Archive (ENA) under BioProject accession number PRJEB38784 and sample accession numbers ERS4649642–ERS4649666. The removal of mitochondrial and chloroplast sequences was performed with R Studio Version 1.2.5, using the ‘phyloseq’ package for microbial sequence analysis. All statistical methods were applied to a final dataset of 32,073 assigned ASVs. In order to account for different sequencing depths, the relative abundances of ASVs within each sample were calculated.

#### 2.2.3. Statistics and Data Visualization

In order to compare the diversity of microbial taxa between plastic debris and reference soil, alpha diversity indices were calculated from the final dataset. Differences in microbial species richness (Chao1), evenness (Pielou) and diversity (Shannon–Weaver and Simpson diversity indices) between plastic debris and reference soil samples were tested using a Wilcoxon rank-sum test. Differential abundance of microbial taxa in plastic debris versus reference soil samples were calculated as the Log_2_ ratio of the difference in mean relative abundance of ASVs on the genus level, where Log_2_FC = Log_2_(Abundance_Plastic_) − Log_2_(Abundance_Soil_). Only genera with a single occurrence higher than 1% relative abundance were chosen for presentation. Positive Log_2_ fold change values indicated a preferential occurrence on plastic debris, whereas taxa that occurred preferentially in the reference soil had negative Log_2_FC values. In order to confirm these results, a differential abundance analysis was performed using the DeSeq2 package in R [37]. To address differences in the microbial community structure, a principal coordinate analysis (PCoA) was performed based on a Bray–Curtis distance matrix that visualized taxonomic dissimilarities of samples in a two-dimensional space as a measure of beta diversity. A PERMANOVA test (*p*-level of significance < 0.05) was used on Bray–Curtis dissimilarities to verify the distinction between the community structure of samples that were either associated with plastic debris or reference soils (‘vegan’ package in R [38]). Bubble plots were generated in R with ggplot2 v.3.1.0 package [39], showing taxonomic abundances across samples. Only ASVs with a relative abundance higher than 0.5% are presented in the plot. All analyses were performed using R version 4.1.0 (18 May 2021).

#### 2.2.4. Thermogravimetric Analysis for Plastic Content Quantification

In order to quantify the amount of plastic in our samples and also account for unapparent microplastic present in the soil, we applied thermogravimetry-mass spectrometry (TGA-MS) as a method for total quantification of all synthetic polymers present in our sampled matrices. For TGA-MS, soil and plastic material was air-dried overnight at 60 °C and 2 mm sieved to remove larger parts from the soil and plastic matrix. Sieves and tools were cleaned and sterilized thoroughly between each handling of the samples. For samples that contained larger plastic particles or foils, the material was homogenized using a cryogenic homogenizer (Retsch CryoMill, Haan, Germany) prior to measuring. TGA-MS measurements were conducted with the thermo-balance TGA/DSC 3+ (Mettler Toledo, Columbus, USA) in 150 μL alumina crucibles from 25 to 600 °C under constant nitrogen flow at a heating rate of 10 K min^−1^. Sample masses varied from 72 to 176 mg depending on the sample composition. The mass content was determined by evaluating the mass loss in the temperature range from ~390 to 515 °C. In this range, decomposition of most common polymers occurs. In addition, measurements were carried out on model samples to exclude any superposition of the mass loss of the organic soil material and the polymers. The model sample was composed of standard soil type 2.3 (organic carbon content 0.66 mass %, LUFA Speyer, Speyer, Germany) and milled peat. Through these tests, it was determined that a ranking of the samples according to their polymer mass content was possible (Supplementary Figure S1).

### 2.3. Colonization Experiment

#### 2.3.1. Plastic Material and UV-Weathering

The plastic material used in the experiments was kindly provided by ‘Bundesanstalt für Materialforschung und prüfung’ (BAM), Berlin, and ‘Plastics Europe Deutschland e.V.’. This study used PE as the plastic material, which was provided in the form of standard chips of 1 cm in diameter and circa 1 mm thickness and as grounded PE powder (particle size 300 µm) for enrichment cultures. In order to mimic microbial surface colonization of PE in the environment, the PE chips were UV-weathered for 812 h in a UV weathering test chamber (Global-UV Test 200, Weiss Umwelttechnik, Vienna, Austria) with UV-fluorescence lamps according to DIN EN ISO 4892-3, type 1A (UVA-340). Additionally, the UV-weathering of the plastic samples was accelerated through exposure of the chips to 60 °C and 85% relative humidity. Plastic chips were placed vertically in metal rails that covered about 2 mm of the chip’s periphery, so that this part remained unaffected by UV radiation. The backsides of the chips were irradiated by the UV beams that readily passed through the transparent chips and got reflected from the mirroring backside of the fixing rail. In this way, plastic samples received a total surface energy of 120 MJ/m^2^, equivalent to 80–160 months outdoor weathering in a temperate European climate. The PE powder used for enrichment cultures was not UV-weathered prior to incubation.

#### 2.3.2. Medium and Preparation of Microbial Inoculum

Microbial slurries were prepared with plastic debris from the visited landfill and incubated in minimal salt medium (MSM) according to the protocol of Daniel Burd (2008) [40]. The medium contained sterilized PE powder as the only carbon source. One liter of carbon-free minimal salt medium contained: 0.1% (1 g/L) (NH_4_)2SO_4_, 0.1% (1 g/L) K_2_HPO_4_, 0.1% (1 g/L) NaNO_3_, 0.1% (1 g/L) KCl, 0.02% (0.2 g/L) MgSO_4_ and 0.01% (0.01 g/L) yeast extract. After the medium had cooled down from autoclaving at 120 °C, 1% 1M cycloheximide was added as a fungicide and 3 g of the sampled material per sampling spot was added to sterilized Schott bottles (50 mL) containing 30 mL of MSM.

To ensure a representative sampling of both planktonic cells and those forming biofilms, the bottles were kept in a sonication bath for 5 min at 30 kHz and 240 W according to Morris, Monier and Jaques (1998) [41]. The slurries were eventually centrifuged for 1 min at 1000× *g* and the supernatant was pipetted off for inoculation. Six UV-weathered PE chips were inoculated with the prepared microbial slurries. The cultures were incubated in closed Schott bottles with 30 mL of MSM + 0.1% cycloheximide and placed in a rotary shaker (100 rpm) at 28 °C in the dark for 102 days.

#### 2.3.3. Light Imaging and Field Emission Scanning Electron Microscopy of Microbial Colonization

For the first direct observation of bacterial colonies and biofilm on the UV-weathered PE chips after 102 days of incubation, three of the PE chips were taken and prepared for light-imaging using a Leica MZ10F stereo microscope. In order to fixate the attached biofilm after incubation, the PE chips were treated with increasing concentrations of ethanol (20, 40, 60, 80, 100%) and dried at room temperature in sterile aluminum foil.

Field emission scanning electron microscopy (FESEM) was used to visualize any UV-induced surface changes of the PE chips and to further investigate the microbial colonization and biofilm attachment on the incubated PE surface. A first FESEM analysis was conducted before and directly after UV-weathering of the PE chips, to detect UV-induced surface changes. One representative PE chip was coated with a ~20 nm carbon layer using a (BalTec Med 020, Leica, Wetzlar, Germany) sputter coater and subsequently imaged using a secondary electron detector at an operating condition of 20 KeV acceleration voltage and 30 µm aperture with a ZEISS Gemini Ultra Plus scanning electron microscope. After exposing the UV-weathered PE chips and an unweathered control to mixed microbial cultures for 102 days, three selected PE chips (two UV-weathered and one control) were analyzed using FESEM in order to visualize any microbial attachment and interaction with the plastic surface. Three selected plastic chips were cleaned of excessive biofilm layers using a 2% sodium dodecyl sulfate (SDS) solution following the protocol of Gilan et al. (2004) [42]. In brief, the plastic chips were shaken in a 2% SDS solution at room temperature for 1 h (200 rpm), then rinsed with distilled water and air dried overnight at 60 °C. Subsequently, these samples were also fixated with increasing concentrations of ethanol and imaged as described above.

## 3. Results

### 3.1. Diversity and Community Composition

A total of 3,272,004 raw reads were obtained from the 25 samples, with an average of 130,880 reads per sample. After merging and quality filtering of the sequencing data, 3.23% of the raw reads were filtered out, leaving a total of 3,166,266 reads with an average of 126,650 reads per sample. We included a total of 32,073 ASVs to calculate different alpha diversity indices of both plastic debris and reference soil.

**Figure 1:**
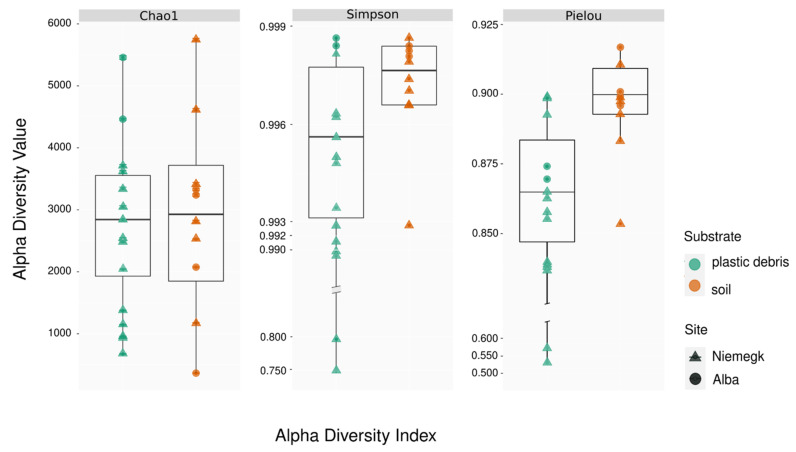
Microbial alpha diversity compared between plastic debris and reference soil (soil) by sampling location (site). The alpha diversity is shown as a measure of the Chao1 index of species richness, the Simpson index of dominance and Pielou’s evenness index. Points represent the calculated diversity measures of the sequenced microbial communities, whereas the boxes span the interquartile range (IQR) of all measures, with the middle line being the median of the calculated indices. A statistical summary is represented in Supplementary Table S1.

According to the calculated alpha-diversity indices, the overall microbial diversity was higher in reference soils than on plastic debris (Figure 1).

The Simpson’s index of diversity (D) indicated that plastic debris samples had a significantly lower microbial diversity than the reference soils (MedianD_plastic_ = 0.995 and MedianD_soil_ = 0.998). The Shannon index of diversity (H) was also found to be significantly lower in the plastic-associated communities than in soil communities (MedianH_plastic_ = 6.76 and MedianH_soil_ = 7.12). Along with the species diversity, the evenness of the bacterial community was also lower in plastic debris than in soil, as shown by Pielou’s evenness index. The Pielou index indicated a significantly lower species evenness in the plastic-associated community (MedianPielou_plastic_ = 0.86) than in the reference soil samples (MedianPielou_soil_ = 0.90) (Supplementary Table S1).

To further investigate the structural differences between the reference soil microbial communities and those inhabiting plastic debris, a principal coordinates analysis (PCoA) of taxonomic community profiles was calculated. We observed a separation along the primary principal coordinate between plastic debris and reference soil samples.

**Figure 2:**
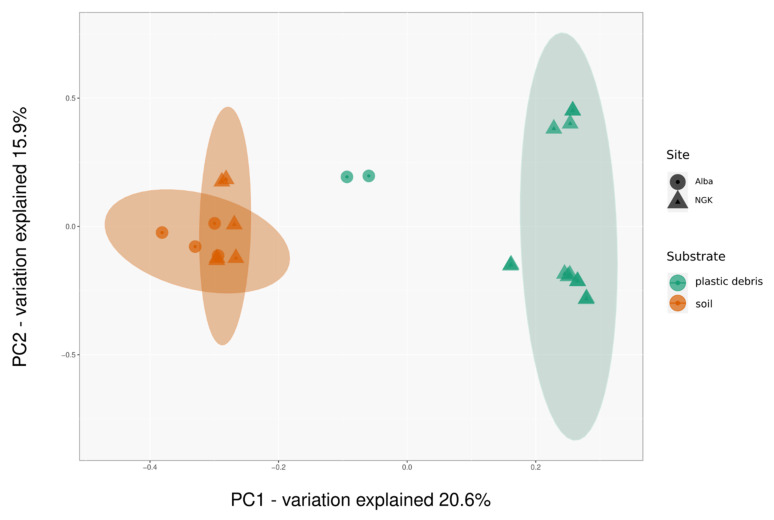
Principal coordinate analysis (PCoA) of distances between microbial communities. Clusters are based on the type of substrate (R^2^ = 0.193) and the two sampling sites, Alba and NGK (R^2^ = 0.08). Plastic debris samples clustered separately from the reference soil samples. The sampling site had a minor effect on the ordination, yet led to a separation of the two plastic Alba samples from the rest of the plastic debris samples. Ordinations are based on Bray–Curtis measures of dissimilarity and ellipses indicate 95% confidence intervals.

Soil samples of both sampling locations clustered together and were clearly separated from all of the plastic debris samples (Figure 2). Testing the multivariate homogeneity of group dispersions, the variances between both groups were found to be equal according to the betadisper function in R with *p* = 0.1555 (Supplementary Table S2).

A two-way PERMANOVA analysis verified that the type of substrate explained 19% of the observed variance between the samples (Supplementary Table S3), meaning that the presence of plastic had a significant effect on the community composition (R^2^ = 0.19347 with *p* < 0.001). A geographical site-effect was observed with samples from the same location, as plastic debris samples from the site Niemegk (NGK) clustered separately from the two samples of the Alba site (Figure 2). However, the effect was less significant for the clustering of samples than the presence of plastic, and sampling site explained only 8% of the observed variance (R^2^ = 0.07992 with *p* < 0.006).

### 3.2. Community Assembly

In order to determine the taxa most responsible for the differences along the primary PCoA axis, the relative abundances of the top 50 most abundant ASVs were compared.

**Figure 3:**
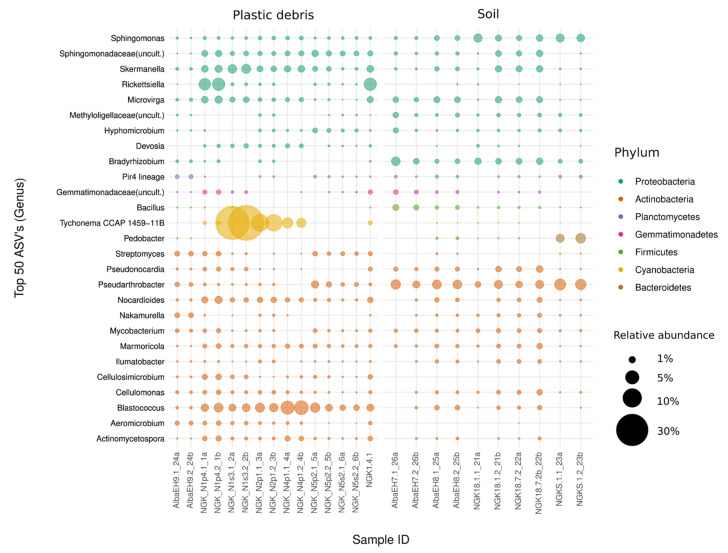
Bubble plot of the top 50 most abundant bacterial ASVs and their corresponding genus present in plastic debris and reference soil (soil). Colors indicate the corresponding phylum of the listed taxa. The size of the bubbles represents their relative abundance.

The most outstanding taxa found in the soil samples belonged to the phyla Actinobacteria (*n* = 13 ASVs) and Proteobacteria (*n* = 9 ASVs). Their most abundant ASVs represented up to 4.6% (genus Pseudarthrobacter), 2.7% (genus Bradyrhizobium), 2.5% (genus Sphingomonas) and 1.5% (genus Microvirga) of all sequenced reads within a given community (Figure 3).

Apart from these, Pedobacter of the phylum Bacteriodetes was among the most abundant ASVs in soil and reached 3.5% relative abundance in a given soil sample (0.6% of mean relative abundance).

Similar to the soil samples, Actinobacteria and Proteobacteria were highly represented in plastic debris. However, the individual abundances differed between the two types of substrate, and the most abundant taxa in plastic debris reached higher abundances than those in the soil overall (Supplementary Figure S2 and Tables S5–S7).

In plastic, the genus Blastococcus of the order Frankiales was the most abundant Actinobacteria, with up to 7% of all total bacterial reads, followed by the Proteobacterium Rickettsiella, with up to 5.8% of the maximum relative abundance, and the genus Skermanella, accounting for up to 3.9% of all sequenced reads in a given plastic community (Supplementary Table S7). However, most remarkable was the fact that the ASVs which reached the highest abundances in plastic debris across both sampling locations were Cyanobacteria of the taxonomic order *Nostocales*. The genus *Tychonema CCAP 1459-11B* occurred in 10 out of 15 plastic debris samples and dominated the community with relative abundances of up to 49.4% of all sequenced reads in the given samples (Figure 3) and accounted for 8.3% of all sequenced reads in plastic debris on average (Supplementary Table S7).

Tychonema CCAP 1459-11B was also present in the reference soil samples but in negligible abundances, with the highest abundance being 0.03% in a given community (on average 0.01% relative abundance in soil). It is noteworthy that none of the dominant taxa in the reference soil samples reached higher relative abundances than 4.6% (genus Pseudarthrobacter) of all reads, whereas the plastic-associated microbial communities were shaped by a few dominant ASVs with high abundances (Supplementary Figure S2).

**Figure 4:**
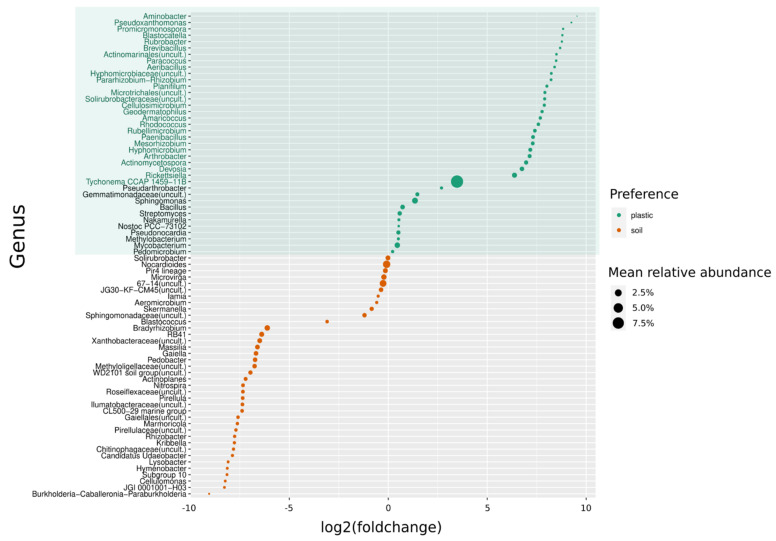
Differential abundance of bacterial genera enriched in plastic debris (plastic) versus reference soil (soil). Taxa with positive Log2 fold change values were more abundant on plastic debris than in the reference soil (green box). Genera names in green occurred exclusively in plastic debris (Log2FC = ∞).

**Figure 5:**
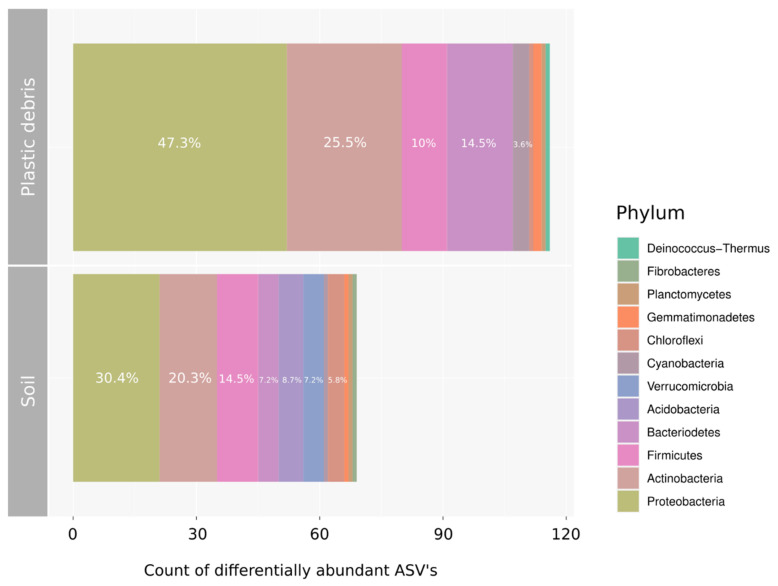
Number and taxonomic composition of differentially abundant ASVs aggregated to phylum level. According to DeSeq2 analysis, a total of 116 ASVs were specific to plastic debris, whereas 69 ASVs could be assigned as being soil-specific. The percentage shows the proportional contribution of the different phyla to the plastic- or soil-specific bacterial community.

Differential abundance analysis (DeSeq) was performed by comparing total abundances of ASVs present in plastic debris and those present in reference soil. By calculating the Log2 fold change values of significantly differing ASVs (*p* < 0.1), bacterial taxa were identified that occurred preferentially (Log2 fold change > 0) and/or exclusively on plastic debris (Figure 4, Supplementary Figure S5). In order to compare the individual mean relative abundance of taxa showing a preference for one of the two substrates, Figure 4 shows ASVs aggregated to the taxonomic level of genus and their average contribution to the sequenced community. A total of 116 ASVs showed positive Log2 fold change values in comparison to 69 ASVs with negative Log2 fold change values (Figure 5, Supplementary Figure S5). Almost half of the ASVs that occurred preferentially on plastic were members of the phylum Proteobacteria (47.3%). A selection of Proteobacteria that occurred exclusively on plastic include *Aminobacter, Pseudoxanthomonas, Paracoccus, Rickettsiella* and *Devosia*, among others (Figure 4).

Actinobacteria was the second most represented phylum, with 25.5% of the plastic specific ASVs, from which genera such as *Actinomycetospora, Arthrobacter, Rhodococcus, Rubrobacter* and *Cellulosimicobium* were among those with higher log2 fold change values. Additionally, the phylum Bacteriodetes was well represented with ASVs enriched on plastic debris (14.5%). Here, members of the family *Flavobacteriaceae* were the most frequent.

*Aeribacillus, Brevibacillus, Planifilum* and *Paenibacillus* were examples of genera of the phylum Firmicutes, to which 10% of all plastic specific taxa belonged, according to our analysis. The order *Nostocales* represented all of the Cyanobacteria ASVs, which, after all, accounted for 3.6% of ASVs enriched on plastic debris.

When aggregated to the level of genus, it became clear that those genera which occurred exclusively on plastic were present in rather low abundances (Figure 4). Only two taxa (*Rieckettsiella: 1.2%* and *Tychonema CCAP 1459-11B: 8.9%*) had mean relative abundances higher than 1 percent. The majority of the plastic-specific genera accounted for a small fraction of the whole community with mean relative abundances ranging between 0.13% (*Aminobacter*), 0.16% (*Pseudoxanthomonas*), 0.24% (*Brevibacillus*), 0.29% (*Aeribacillus*), 0.38% (*Planifilum*), 0.59% (*Rhodococcus*) and 0.92% (*Devosia*). Similar to the bacterial ASVs accounting for the plastic-specific community, most ASVs which occurred preferentially in soil samples belonged to the phylum of Proteobacteria (30.4%). Ten out of twenty-one of ASVs from the Proteobacteria belonged to the order of *Rhizobiales,* followed by *Betaproteobacteriales* and *Myxococcales.*

The second most represented phylum of ASVs enriched in soil samples was Actinobacteria (20.3%). Firmicutes accounted for 14.5% of the soil specific ASVs, of which the majority belonged to the order *Clostridiales* and *Bacillales.* The other well-represented phyla were Acidobacteria (8.7%), Chloroflexi (5.8%) and Verrucomicrobia (7.2%). Additionally, the phylum Bacteriodetes was well-represented among the soil-specific taxa (7.2%); however, it was only half as enriched in soil as members of Bacteriodetes were in plastic debris (14.5%) (Figure 5, Supplementary Table S7). The mean abundances of the taxa enriched in soil were overall slightly higher than those of the plastic-specific genera. The rarest genus was *Burkholderia-Caballeronia-Paraburkholderia*, with 0.18% of the mean relative abundance, followed by the genus JGI 0001001-H03, with 0.32%, *Cellulomonas* (0.33%), *Rhizobacter* (0.46%), *Nitrospira* (0.61%), *Gaiella* (0.97%), Xanthobacteraceae (uncult.) (1.1%), RB4*1* (1.2%), and *Nocardioides* was the most abundant genus, with 2.9% of mean relative abundance (Figure 4).

### 3.3. Surface Deterioration of Polyethylene Facilitated by UV-Weathering and Microbial Attachment

**Figure 6:**
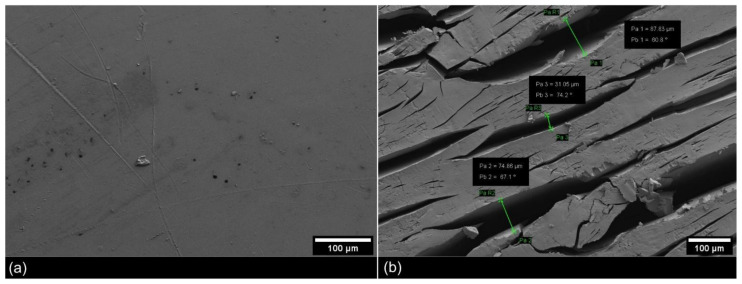
FESEM images of PE chips before and after UV-weathering. Images show the surface of an (**a**) unweathered PE chip and (**b**) the surface changes after 812 h of UV radiation at elevated temperature (60 °C) and 85% relative humidity.

**Figure 7:**
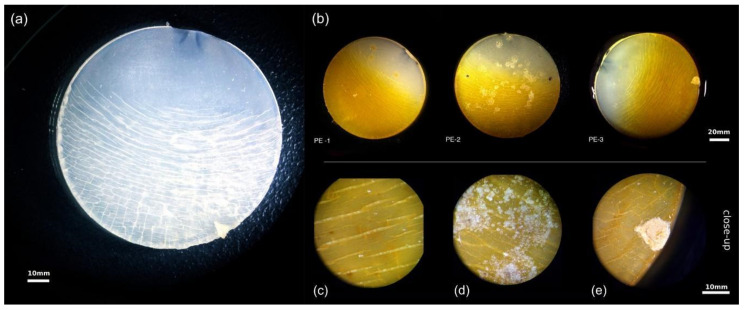
Light microscopy images of PE chips before (**a**) and after 102 days of incubation in batch cultures inoculated with microbial communities from the landfill (**b**). The PE samples show biofilm formations on the surface and inside cracks (**c**). White bacterial aggregates were visible (**d**), and one sample had a firmly attached white colony (**e**). Black dots are pencil marks for orientation.

The FESEM analysis revealed a clear change in the texture of the PE surface due to the UV-weathering treatment (Figure 6 and Figure 7a). The UV-weathered PE surfaces developed many cracks relative to the unweathered PE samples, which showed no indication for surface damage. Form and sizes of the cracks varied and ranged from a few micrometers up to around 100 microns in width.

An inspection of the incubated UV-weathered PE chips with a light-microscopical binocular showed patches of white colonies that covered the PE surface (Figure 7b) and small rounded aggregates occupying most of the larger cracks (Figure 7c). The biofilms closely adhered to the PE surface and scraping off was difficult. Most apparent were rounded bacterial aggregates that filled the larger cracks of the polymer. They occurred in all studied PE samples, and were detectable even at the macroscopic scale as bulky white colonies (Figure 7d,e).

**Figure 8:**
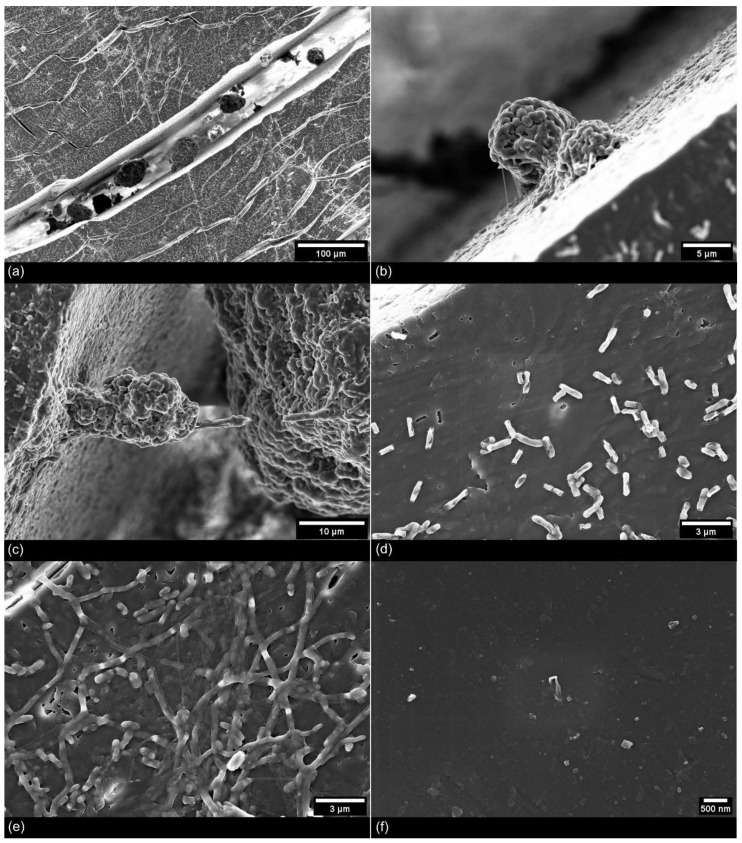
FESEM images of mixed microbial biofilms on PE chips after 102 days of incubation. (**a**–**c**) Rounded aggregates of bacterial cells were found inside the surface cracks and tightly attached with strings of EPS matrix. (**d**) Singular rod-shaped bacteria attached to the surface, adjacent holes resembled bacterial contours. (**e**) Bacterial cells attached to the surface in a network-like structure. (**f**) Untreated PE chips with poor bacterial cell attachment after incubation.

After the incubation in liquid cultures, a microbial biofilm developed mainly in surface grooves and cracks of the UV-weathered PE chips, while noticeably less biofilm formation was observed on the unweathered chips (Figure 8f).

Nevertheless, bacteria were also visible on the surface of the unweathered PE chips, but their distribution was more scattered and the colonies did not develop a dense biofilm. When further analyzed with FESEM, these aggregates were highly abundant inside micrometer-sized surface cracks, where they showed distinct morphologies: they formed large (ca. 5–50 µm), rounded structures with an irregular surface that suggested a mixture of bacterial cells surrounded by EPS (Figure 8a–c). A close-up of the mentioned aggregates revealed a large number of single rod-shaped bacterial cells that were embedded in EPS. The surrounding matrix gave shape to the aggregates by forming cross-connections and threadlike structures inside the biofilm.

The observed strings of EPS attached the aggregate to the polymer surface (Figure 8b–c). In general, due to their rather voluminous shape, the direct contact with the plastic surface seemed limited to a small fraction of the biofilm community. Strings of EPS were easily visible between single aggregates and between aggregates and the plastic.

Most striking were the highly abundant rod-shaped cells detected on the UV-weathered PE surface which seemed to closely interact with the substrate. All the cells were individually attached to the surface with string-like structures, and in their closer proximity, irregular shaped holes and pits appeared on the plastic surface (Figure 8d). Those holes were visibly distinct from the other cracks in both their shape and orientation, as cracks formed by UV-weathering followed the direction of original polymer extrusion. In some cases, the holes resembled the shape of the neighboring bacterial cells, which suggested a recent attachment and a visible disintegration of the polymer substrate at this very spot (Figure 8d). These holes were not present on UV-weathered PE without bacterial inoculation (Figure 6b). Finally, small irregularly rod-shaped bacteria closely adhered to the PE surface and were organized in structures that resembled networks of EPS (Figure 8e).

## 4. Discussion

Our study suggests that in response to the presence of plastic as a physical substrate of growth and potential carbon-source, plastic debris hosts a distinct microbial community with a reduced species diversity (evenness and richness) relative to soils without a significant amount of plastic from the same geographical location.

A reduced community evenness was reported to be accompanied by an increase in a few taxa in response to environmental conditions favoring their abundance relative to other taxa [14]. In our study, those bacteria that dominated the plastic debris samples accounted for large fractions of the community and were significantly more abundant than the most dominant taxa in the reference soils. A similar trend was observed by Huang and colleagues (2021) on submerged LDPE fragments which were incubated 90 days in soil [16]. In their study, the microbial community on plastic debris became significantly less even over the course of the incubation. Additionally, Zhang and colleagues observed a reduced alpha diversity on terrestrial macroplastic of mulching foils when compared to the surrounding soil [15].

Likewise, our principal coordinate analysis (PCoA) showed that 19% of the differences in community structure could be explained by the presence of plastic in the samples. At the level of community composition, the majority of bacterial taxa were shared between plastic- and reference soil samples, but their relative contribution to the community differed significantly. Although we observed a distinct microbial community relative to soils absent of plastic debris, those taxa that are known to degrade plastic, such as *Rhodococcus* sp. or *Pseudomonas* sp. [25,38], were only present in low abundances (less than 1% relative abundance).

This is different from recent discoveries suggesting that the presence of plastics leads to an enrichment of microorganisms which possess plastic-degrading abilities [16,22]. An exciting example of such studies is the discovery that *Ideonella sakaiensis* obtained from plastic debris that can degrade polyethylenterephthalat (PET). In a study from 2019, Zhang and colleagues also suggested plastic fragments in soil to serve as a ’special microbial accumulator’ enriching plastic-degrading microorganisms [15]. Here, the plastic-associated microbial community was rather dominated by biofilm-forming autotrophic bacteria tolerant to hydrophobic environments and in particular those samples with the lowest evenness showed a high dominance of cyanobacteria of the taxonomic order Nostocales. Nostocales are photo-autotrophic bacteria that inhabit upper soil layers and are often pioneer species in ecosystems degraded due to anthropogenic activities [43]. They were identified as inhabitants of heavily petroleum-polluted microbial mats, and other studies have even described their capacity to degrade hydrocarbons after oil spills [43,44]. The samples that were dominated by these cyanobacteria contained mixed plastic debris with a dark organic matrix attached to them, which at closer observation appeared as a microbial mat or biological soil crust (biocrust). Cyanobacteria in general have the capacity to promote biocrust formation and increase soil fertility by contributing to the positive net-production on plastic debris as a substrate of growth [45]. Their ability to fixate nitrogen and perform photosynthesis makes them important symbionts for other aerobic heterotrophic bacteria in nutrient scarce surfaces such as synthetic polymers. Abed and Köster (2005) hypothesized that cyanobacteria provide the nutrients for other hydrocarbon-degrading species, much more than the amount of hydrocarbons they actually degrade themselves [46]. Their resilience to hydrocarbon polluted sites and their role as possible nutrient providers on otherwise nutrient scarce polymers suggests them as important functional inhabitants of a potential ‘terrestrial plastisphere’. We, therefore, suggest that the high abundance of Nostocales in some of the plastic debris samples is due to their ability in being pioneering taxa in early biocrust succession.

Besides cyanobacteria, other taxa such as *Geodermatophilaceae*, *Nocardioides*, *Beijerinckiaceae* and *Rubrobacteriaceae* were found to be abundant during early biocrust stages in degraded soil systems [47] and were present on plastic debris in our study as well. The potential role of these biofilm inhabitants in the biodegradation of plastic debris is not well understood and should be further investigated.

Still, our findings suggest that plastic debris in soil forms a habitat in which, at an early stage of soil development, nutrient availability is secured by pioneering autotrophic bacteria tolerant to hydrophobic environments. This appears reasonable also because the plastic-derived carbon in PE and other plastics of high molecular weight is not readily available for microbial utilization. Their hydrophobic carbon–carbon backbone and the lack of functional groups make them resilient against biodegradation and create a microbial niche depleted of bio-available carbon and other essential nutrients like nitrogen and phosphorous. This effect of nutrient limitation by nitrogen and phosphorous has been shown in hydrocarbon-polluted ecosystems during microbial oil-spill bioremediation [48]. A high content of hydrophobic substances creates a selective stressor for microbial communities, and it can be assumed that a high plastic content in soil will have a similar selective effect on the microbial community [49]. Different metabolic strategies and synergetic effects will be essential in such nutrient scarce ecosystems. This means that taxa capable of either directly utilizing plastic-derived carbon and taxa that carry out important secondary functions within the interactive network should dominate the system.

Although in our study, known plastic-degrading microorganisms could not be directly identified among the most abundant taxa based on the molecular approach chosen, there may still be plastic-degrading taxa among them, since many microorganisms that were initially not considered to be hydrocarbon-degrading bacteria were found to contain genes homologous to alkane-hydroxylase genes essential for enzymatic hydrocarbon degradation [50]. Among these were taxa such as *Mycobacterium, Brevibacterium, Burkholderia* and *Nocardioides*, which were all abundant in the plastic-associated community of this study and are thus potentially involved in breaking-down the hydrocarbon backbone of plastics. However, our study shows limitations regarding the specific metabolic niches of taxa observed to be associated with plastic debris in soil because of the difficulty experienced in conclusively finding plastic-degrading properties from taxonomic data only [24,51]. A metagenomic approach would reveal enzymatic pathways and genes enriched in plastic-associated microbial communities in soil.

Regardless of the microbial taxa present, the degradation of plastics by microorganisms requires that the organisms possess cellular properties that allow an interaction with the hydrophobic plastic surface [52]. For this reason, we studied plastic-associated communities with regard to their surface adhesion to UV-weathered PE chips and used FESEM showing that pretreated PE experienced a severe growth of bacterial colonies and a dense colonization inside the surface cracks after 100 days of in vitro incubation. This means that the artificial weathering prior to incubation was successful in facilitating attachment and biofilm formation of microbes onto the PE surface, and therefore paved the way for a potentially further successive biodegradation to take place. Additionally, Weinstein and colleagues (2016) showed that the colonization of various plastic types by a dense microbial biofilm resulted in the production of microplastic intermediates after 8 weeks of incubation in the field [53].

For long-chained polymers such as PE that are characterized by a carbon–carbon backbone and high molecular weight, biodegradation occurs as a result of abiotic weathering and biotic interaction [54]. Photochemical weathering acts on the molecular properties of the polymer, oxidizing the polymer surface and introducing hydrophilic groups which enable the first attachment of microbes to the surface [54]. By using FESEM in this study, it was possible to visualize the effects of abiotic weathering on the plastic integrity and the colonization of these UV-induced cracks and fractures at a high-magnification. Even though several studies have used SEM imaging to explore soil-microbial degradation of PE [30,42,55,56,57,58,59], none of them have shown the surface colonization of plastic by mixed microbial cultures with such high-magnification imaging techniques. Our FESEM images show a dense colonization by bacterial cells embedded into a larger extracellular matrix that was firmly attached to the PE surface. The 3D structure of the plastic-associated biofilm became visible and revealed the internal arrangement of bacterial cells connected by strings of EPS and reached down to single cell representation of a few hundred nanometers. Similarly, detailed images were so far only shown in studies using isolated bacterial strains [56] and mixed microbial consortia of aquatic origin [3].

Among the PE chips that showed traces of fragmentation, such as irregularly fringed cracks, were some on which microbial cells appeared to have left holes in the surface resembling their cellular silhouette following strong attachment to the surface of the chips. In a study on the degradation of PE by *Bacillus amyloliquefaciens*, very similar marks of degradation were detected in places where bacteria had initially attached to the plastic [56]. In other studies, microcracks and pitting of plastic surfaces was observed as a result of microbial enzymatic attack of UV-weathered PE [59]. Next to abiotic factors that accelerate the surface fragmentation of plastics, two microbial enzymes were shown to cleave the C-C bond of PE. Both the extracellular laccase from *Rhodococcus ruber* and the alkane hydroxylase of an alkane-consuming *Pseudomonas* strain sp. E4 significantly lowered the molecular weight of PE by oxidizing the polymer chain [60,61]. This shows that PE similar to PET can be at least partially degraded by microbial enzymatic activity. The high resolution FESEM images shown in this study provide further evidence for this. The images show in detail that the PE chips incubated with the *Pseudomonas* sp. E4 strain had traces of surface erosion that were very similar to the traces we found on our PE chips after 100 days of incubation. Therefore, even though we did not measure any physio-chemical changes of the incubated polymer itself, it appears plausible that the various bacterial assemblages observed on the PE surface did not only colonize its surface but also contributed to a disintegration of the polymer and a fragmentation of PE into smaller plastic particles.

## 5. Conclusions

The ecological approach of our study revealed the presence of a distinct microbial community of plastic debris characterized by a few dominant taxa which seem to perform better in the presence of plastic than others and which are known to be important for biocrust formation. Their potential involvement in the degradation of the hydrocarbon backbone of plastic compounds remains, however, unresolved and, despite some evidence for it, the hypothesis of plastic debris as an enriching habitat of plastic degrading taxa could not be directly supported here. Nevertheless, our study aligns with other work showing that bacteria specific to plastic debris rather belong to the rare biosphere [62] and that the biodegradation of plastics is a complex and successional process which often involves the presence of a diverse and multispecies consortium of microorganisms including autotrophs providing nutrients to other members of the consortium which may eventually break down the plastic compounds. Considering our study in light of previous work, we can confirm the existence of the terrestrial plastisphere. Due to the complexity and niche diversity within the degradation of long-chained polymers like PE and based on our work, it must be assumed that different microorganisms are responsible for the various steps of fragmentation and deterioration of the plastic material up to the assimilation and mineralization of plastic-derived carbon [63]. Even though single species of microorganisms were shown to adapt their metabolic system towards the utilization of plastic-derived carbon before, we suggest strengthening research on the degradation potential of microbial communities rather than of single species based on our work. Therefore, the need to better understand the underlying mechanisms and taxa of the microbial attack within such a diverse microbial assemblage is evident. Follow-up research on a suggested ‘terrestrial plastisphere’ should evidence the conversion of the plastic’s carbon into microbial biomass by the use of carbon isotope-labeled plastics and should use fluorophore-labelled oligonucleotides to identify taxa among the terrestrial plastic-associated microbial communities responsible for this conversion.

## Data Availability

Sequencing raw data is publicly available via the European Nucleotide Archive (ENA) under the BioProject accession number PRJEB38784 and sample accession numbers ERS4649642–ERS4649666.

## Supplementary Materials

The following are available online at https://www.mdpi.com/article/10.3390/microorganisms9091876/s1, Figure S1: TGA quantification of the total plastic content in all sequenced samples. Figure S2: Sampling spots of plastic debris and natural reference soil used in our study., Figure S3: Sampled material used for DNA extraction and TGA plastic quantification., Figure S4: Relative abundances of top ten most abundant ASV’s per sample type (plastic debris vs. soil)., Figure S5: Differential abundance of bacterial ASV’s., Table S1: Alpha diversity indices (mean values) of the microbial taxa present in plastic debris (plastic) and reference soil (soil) samples, Table S2: Testing homogeneity of multivariate group variances (soil versus plastic), Table S3: Two-way PERMANOVA (adonis), Table S4: Wilcoxon rank sum test for differences in abundances of Top50 ASV’s plastic debris versus soil, Table S5: Average relative abundances of Top50 ASV’s and corresponding genera in soil, Table S6: Average relative abundances of Top50 ASV’s and corresponding genera in plastic debris, Table S7: Raw relative abundances of Top50 ASV’s.
