# Supplementary material for: The Terrestrial Plastisphere: Diversity and Polymer-Colonizing Potential of Plastic-Associated Microbial Communities in Soil
Source: Microorganisms. 2021 Sep 3;9(9):1876. doi: 10.3390/microorganisms9091876 (PMC8465064; doi:10.3390/microorganisms9091876)
Supplement: Supplementary file 1 [file microorganisms-09-01876-s001.zip › microorganisms-1346230-supplementary/microorganisms-1346230-supplementary-to conversion/Supplementary_FigureS5.pdf]

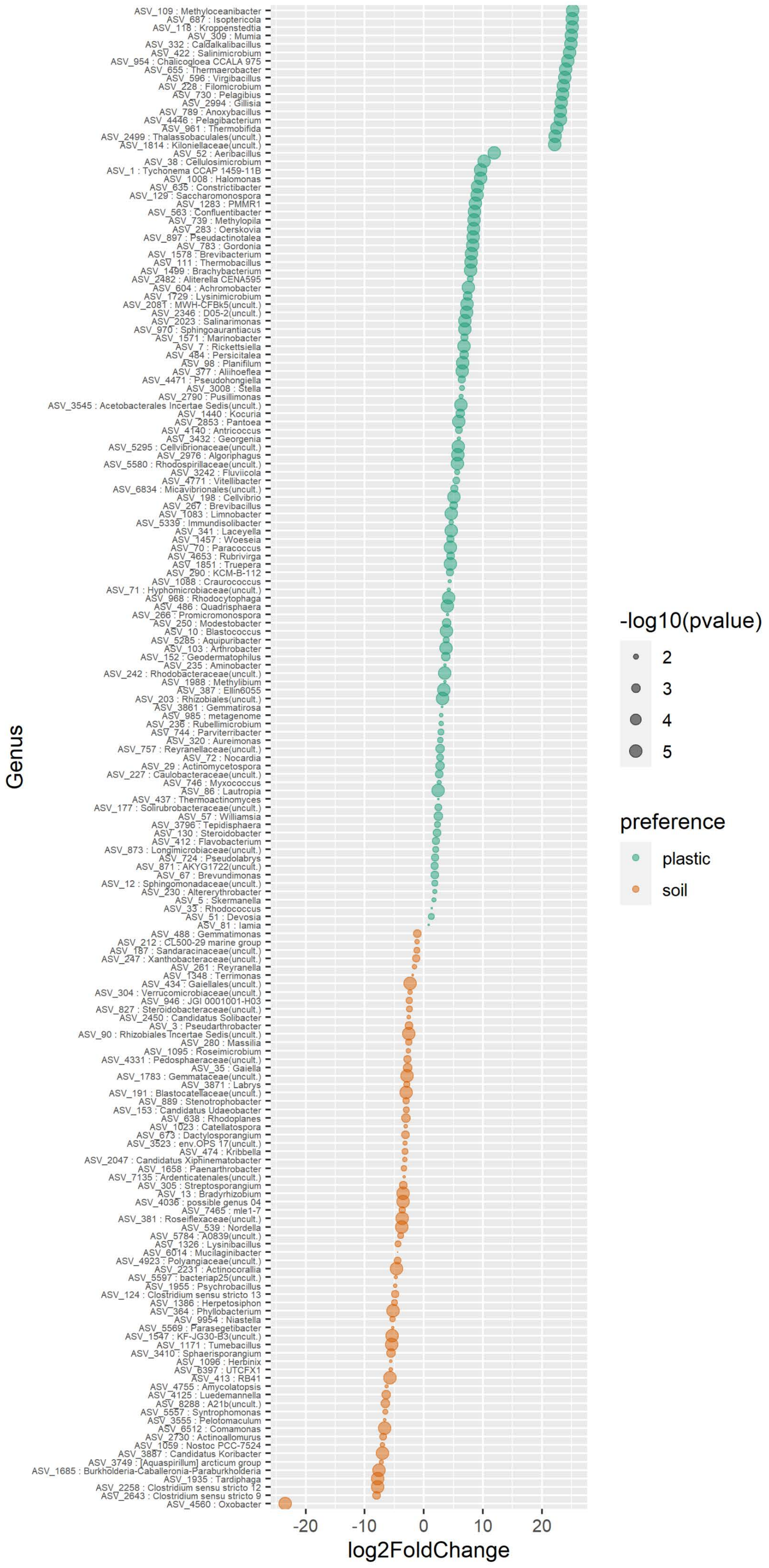

Figure S5: Differential abundance of bacterial ASV's enriched in plastic debris (plastic) versus reference soil (soil). Taxa with positive Log2 fold change values were more abundant on plastic debris than in the reference soil (green box). DeSeq analysis was performed on ASV level and grouped according to their corresponding genus. Significance is given in -log10(p), where larger values indicate a more significant association.
