# Supplementary material for: The Terrestrial Plastisphere: Diversity and Polymer-Colonizing Potential of Plastic-Associated Microbial Communities in Soil
Source: Microorganisms. 2021 Sep 3;9(9):1876. doi: 10.3390/microorganisms9091876 (PMC8465064; doi:10.3390/microorganisms9091876)
Supplement: Supplementary file 1 [file microorganisms-09-01876-s001.zip › microorganisms-1346230-supplementary/microorganisms-1346230-supplementary-to conversion/Supplementary_v8.pdf]

Supplementary

MacLean et *al., The Terrestrial Plastisphere: Diversity and Polymer-colonizing Potential of Plastic-associated Microbial Communities in Soil (2021)*

Table S1. Alpha diversity indices (mean values) of the microbial taxa present in plastic debris (plastic) and reference soil (soil) samples. Significant P values ($p < 0.05$) of the Wilcoxon rank sum test are marked with *.

	Chao1	+/- SD	Wilcoxon Rank sum test (P<0.05)
Plastic	2578.30	1404.99	0.53
Soil	2730.57	1451.21	
Site (NGK vs. Alba)			0.47

	Simpson	+/- SD	Wilcoxon Rank sum test (P<0.05)
Plastic	0.970	0.070	0.012 *
Soil	0.980	0.060	
Site (NGK vs. Alba)			0.014*

	Shannon	+/- SD	Wilcoxon Rank sum test (P<0.05)
Plastic	6.31	1.04	0.03 *
Soil	6.56	0.95	
Site (NGK vs. Alba)			0.08 *

	Pielou	+/- SD	Wilcoxon Rank sum test (P<0.05)
Plastic	0.82	0.11	0.00053 *
Soil	0.85	0.09	
Site (NGK vs. Alba)			0.021 *

Table S2. Testing homogeneity of multivariate group variances (soil versus plastic)

	**Df**	**Sum Sq**	**Mean Sq**	**F value**	**Pr (>F)**
**Groups**	1	0.017161	0.0171615	2.1563	0.1555
**Residuals**	23	0.183047	0.0079586		

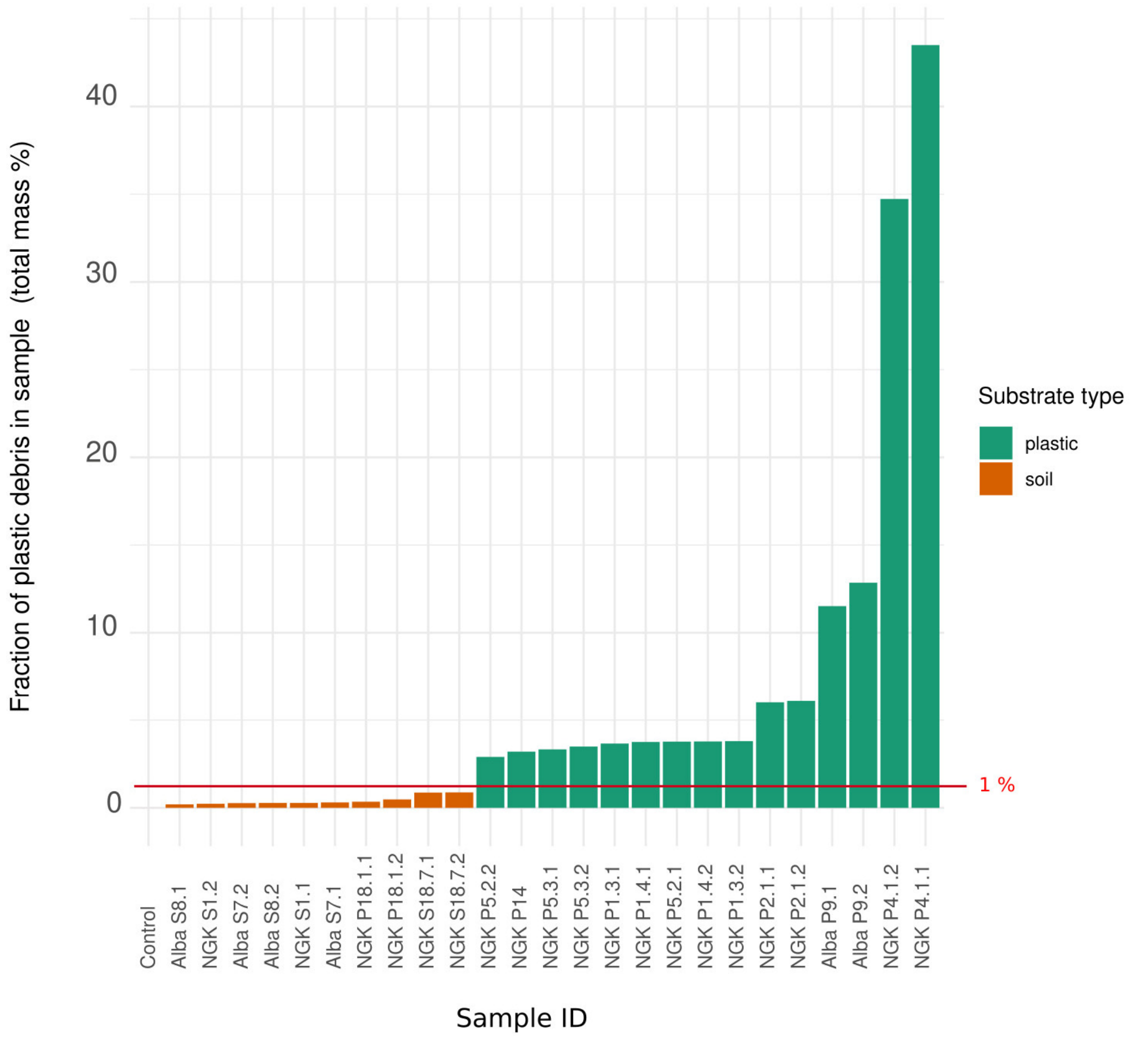

Figure S1. TGA quantification of the total plastic content in all sequenced samples. The content is given in mass percentage of the total (soil) material. Samples which contained less than 1% plastic debris, where considered as natural soil samples without a major influence of plastic on the soil microbial habitat.

Table S3. Two-way PERMANOVA (adonis)

Permutation test for adonis under reduced model
Terms added sequentially (first to last)
Permutation: free
Number of permutations: 999

	Df	SumOfSqs	R2	F	Pr(>F)
**Sediment_type**	1	1.6019	**0.19347**	6.3992	0.001 ***
**Site**	1	0.6617	**0.07992**	2.6433	0.006 **
**Sediment_type:Site**	1	0.7596	**0.09173**	3.0342	0.001 ***
Residual	21	5.2570	0.63489		
Total	24	8.2802	1.00000		

Signif. codes: 0 ‘***’ 0.001 ‘**’ 0.01 ‘*’ 0.05 ‘.’ 0.1 ‘ ’ 1

Table S4. Wilcoxon rank sum test for differences in abundances of Top50 ASV’s plastic debris versus soil

Wilcoxon rank sum test with continuity correction

Abundance by Substrate_type
W = 44750
p-value = 0.001347 *
alternative hypothesis: true location shift is not equal to 0

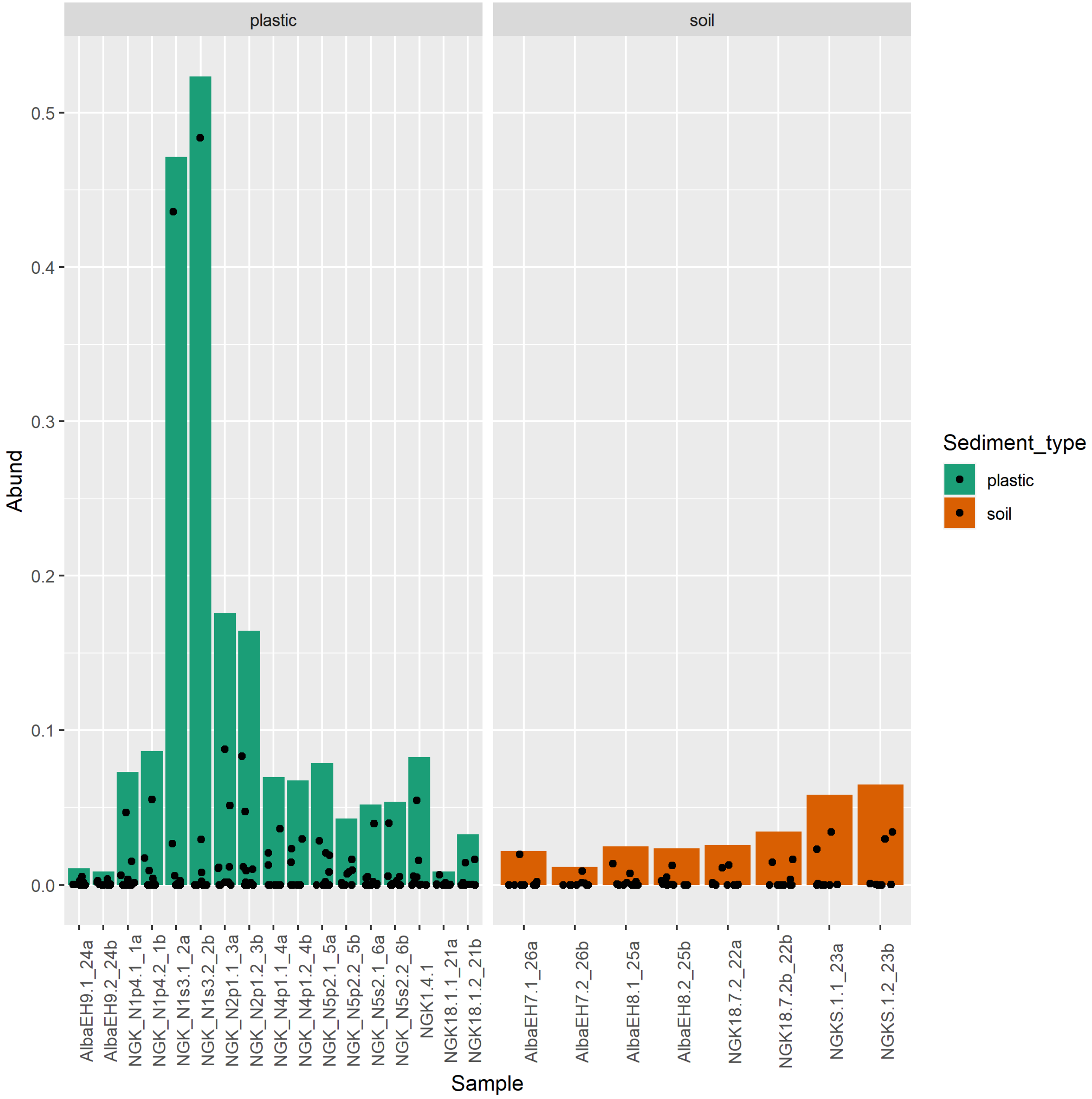

Figure S2. Relative abundances of top ten most abundant ASV’s per sample type (plastic debris vs. soil).

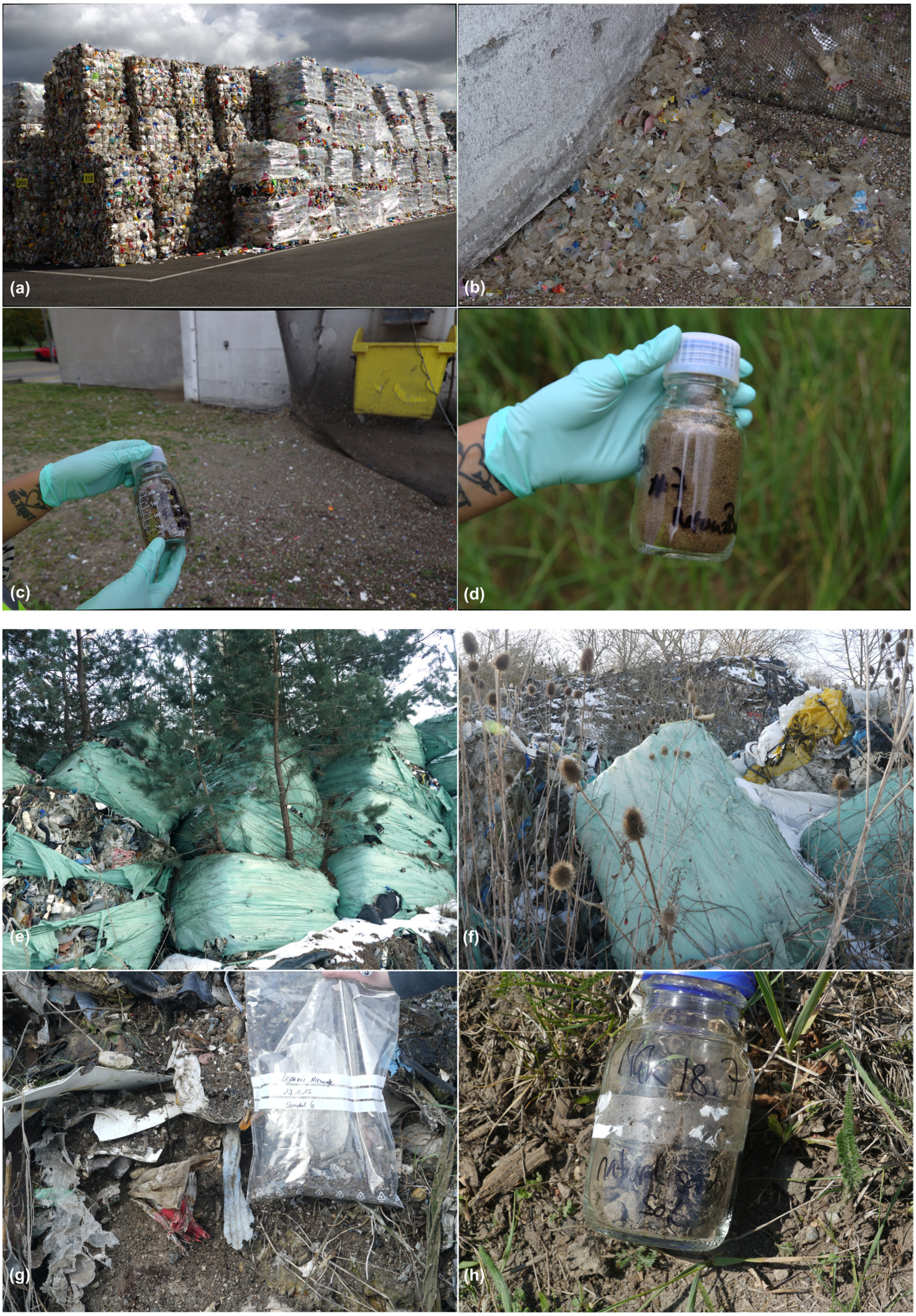

Figure S3. Sampling spots of plastic debris (a-c and e-g) and natural reference soil (d and h) used in our study. Images show the sampling location “Alba” (a-d): a plastic recycling factory and its surrounding terrain (d), as well as the location “Niemegk” (e-h): an abandoned plastic landfill and the adjacent meadow soil (h).

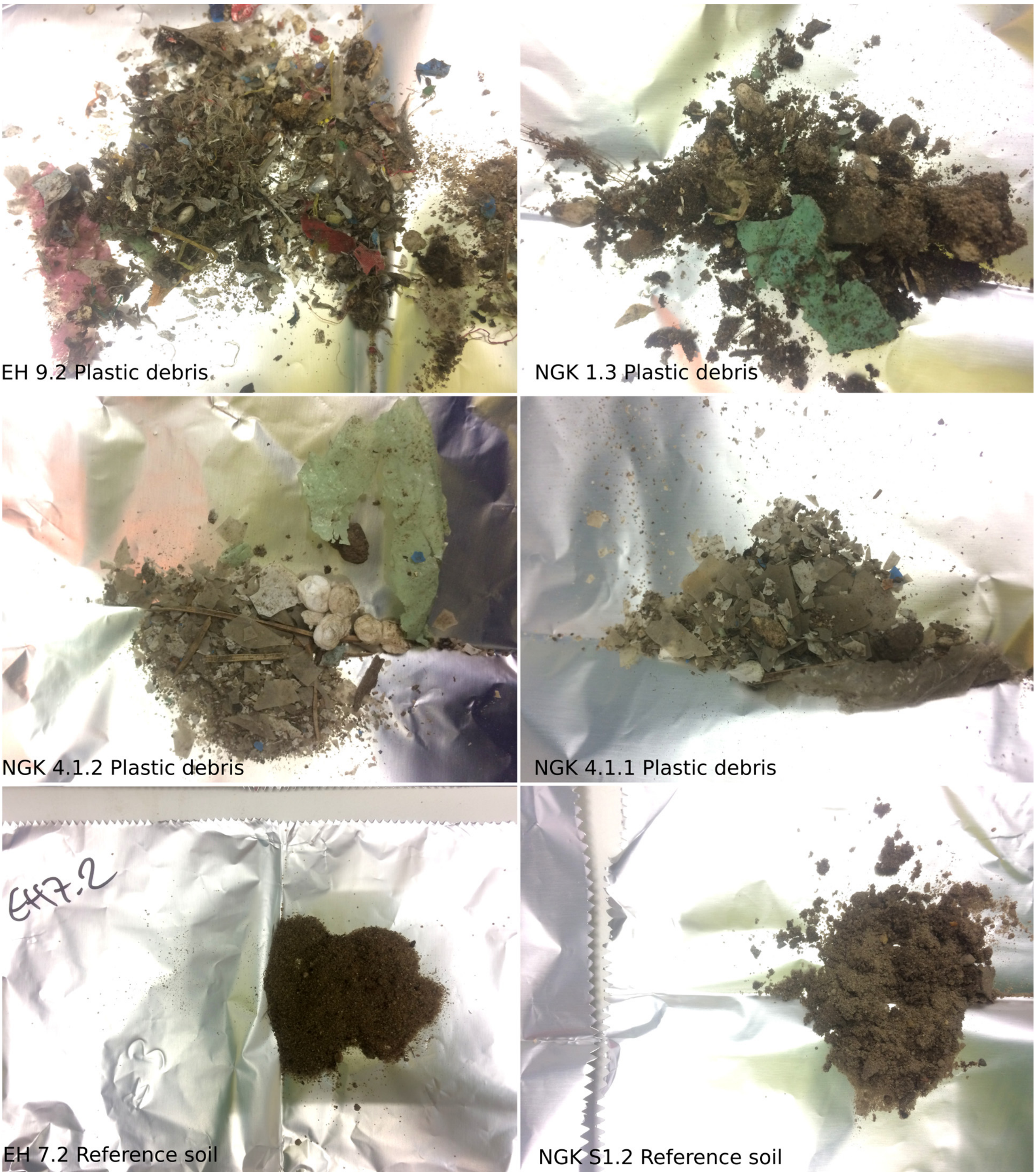

Figure S4. Sampled material used for DNA extraction and TGA plastic quantification. Soil or plastic debris was ground and homogenized using FastPrep ® Bead Tubes inside a FastPrep ® Cell disrupter for DNA extraction and the cryogenic homogenizer Retsch CryoMill for TGA measurements. Larger particles (foils) were cut prior to disruption.

Table S5. Average relative abundances of Top50 ASV's and corresponding genera in soil

Abundances of top 50 ASV's (Genera) in soil					
**Genus**	**N**	**mean**	**sd**	**med**	**se**
**Pseudarthrobacter**	10	0.027	0.011	0.026	0.003
**Sphingomonas**	10	0.019	0.009	0.018	0.003
**Bradyrhizobium**	10	0.012	0.007	0.012	0.002
**Bacillus**	10	0.009	0.006	0.008	0.002
**Microvirga**	10	0.007	0.005	0.010	0.002
**Pedobacter**	10	0.006	0.012	0.000	0.004
**Skermanella**	10	0.006	0.006	0.004	0.002
**Pseudonocardia**	10	0.006	0.005	0.006	0.002
**Mycobacterium**	10	0.005	0.002	0.005	0.001
**Nocardioides**	10	0.004	0.004	0.003	0.001
**Methyloligellaceae(uncult.)**	10	0.004	0.003	0.004	0.001
**67-14(uncult.)**	10	0.004	0.004	0.003	0.001
**Marmoricola**	10	0.004	0.003	0.003	0.001
**Sphingomonadaceae(uncult.)**	10	0.004	0.003	0.003	0.001
**Roseiflexaceae(uncult.)**	10	0.003	0.006	0.000	0.002
**Gaiella**	10	0.003	0.002	0.002	0.001
**Hyphomicrobium**	10	0.003	0.002	0.002	0.001
**Ilumatobacter**	10	0.002	0.002	0.002	0.001
**Pedomicrobium**	10	0.002	0.002	0.001	0.001
**CL500-29 marine group**	10	0.002	0.002	0.002	0.001
**Streptomyces**	10	0.002	0.002	0.002	0.001
**Nakamurella**	10	0.002	0.002	0.002	0.001
**Gemmatimonadaceae(uncult.)**	10	0.002	0.002	0.001	0.001
**RB41**	10	0.002	0.003	0.001	0.001
**MND1**	10	0.002	0.003	0.001	0.001
**Average**		**0.006**			

Table S6. Average relative abundances of Top50 ASV's and corresponding genera in plastic debris

Abundances of top 50 ASV's (Genera) on plastic

Genus	N	mean	sd	med	se
**Tychonema CCAP 1459-11B**	15	0.083	0.162	0.004	0.042
**Blastococcus**	15	0.027	0.021	0.024	0.005
**Skermanella**	15	0.012	0.009	0.012	0.002
**Rickettsiella**	15	0.012	0.022	0.002	0.006
**Sphingomonadaceae(uncult.)**	15	0.010	0.007	0.012	0.002
**Nocardioides**	15	0.009	0.005	0.008	0.001
**Microvirga**	15	0.008	0.007	0.008	0.002
**Streptomyces**	15	0.004	0.003	0.005	0.001
**Marmoricola**	15	0.004	0.002	0.004	0.001
**Actinomycetospora**	15	0.004	0.003	0.003	0.001
**Hyphomicrobium**	15	0.004	0.004	0.003	0.001
**67-14(uncult.)**	15	0.004	0.006	0.001	0.002
**Pseudarthrobacter**	15	0.004	0.005	0.002	0.001
**Cellulosimicrobium**	15	0.003	0.004	0.002	0.001
**Aeromicrobium**	15	0.003	0.002	0.003	0.001
**Devosia**	15	0.002	0.002	0.002	0.001
**Geodermatophilus**	15	0.002	0.006	0.000	0.002
**Arthrobacter**	15	0.002	0.002	0.001	0.001
**Paracoccus**	15	0.002	0.003	0.000	0.001
**Pseudonocardia**	15	0.002	0.003	0.001	0.001
**Methyloligellaceae(uncult.)**	15	0.002	0.002	0.002	0.000
**Rubrobacter**	15	0.002	0.003	0.001	0.001
**Methylobacterium**	15	0.002	0.002	0.001	0.001
**Hyphomicrobiaceae(uncult.)**	15	0.002	0.003	0.000	0.001
**Planifilum**	15	0.002	0.003	0.000	0.001
**Gemmatimonadaceae(uncult.)**	15	0.002	0.002	0.000	0.001
**Aeribacillus**	15	0.002	0.003	0.000	0.001
**Rhodococcus**	15	0.002	0.003	0.000	0.001
**Average**		**0.008**			
